# Angiotensin II induces skin fibrosis: a novel mouse model of dermal fibrosis

**DOI:** 10.1186/ar4028

**Published:** 2012-08-22

**Authors:** Lukasz Stawski, Rong Han, Andreea M Bujor, Maria Trojanowska

**Affiliations:** 1 Arthritis Center, Boston University School of Medicine, Boston, MA 02118, USA

## Abstract

**Introduction:**

Systemic sclerosis (SSc) is an autoimmune inflammatory disorder of unknown etiology characterized by fibrosis of the skin and internal organs. Ang II (angiotensin II), a vasoconstrictive peptide, is a well-known inducer of kidney, heart, and liver fibrosis. The goal of this study was to investigate the profibrotic potential of Ang II in the mouse skin.

**Methods:**

Ang II was administered by subcutaneous osmotic mini pumps to C57BL/6 male mice. Collagen-content measurements were performed with Gomori Trichrome staining and hydroxyproline assay. The mRNA expression level of collagens, *TGF-β1*, *TGF-β2*, *TGF-β3*, *CTGF*, *αSMA*, *CD3*, *Emr1*, *CD45/B220*, *MCP1*, and *FSP1 *were quantified with real-time polymerase chain reaction (PCR). Immunostaining was performed for markers of inflammation and fibrosis, including, phospho-Smad2, αSMA, CD3, Mac3, CD45/B220, and CD163B. Fibrocytes were identified by double staining with CD45/FSP1 and CD45/PH4. Endothelial cells undergoing endothelial-to-mesenchymal transition (EndoMT) were identified by double staining with VE-cadherin/FSP1.

**Results:**

Ang II-infused mice develop prominent dermal fibrosis in the area proximal to the pump, as shown by increased collagen and *CTGF *mRNA levels, increased hydroxyproline content, and more tightly packed collagen fibers. In addition, elevated mRNA levels of *TGF-β2 *and *TGF-β3 *along with increased expression of pSmad2 were observed in the skin of Ang II-treated mice. Dermal fibrosis was accompanied by an increased number of infiltrating fibrocytes, and an increased number of αSMA-positive cells, as well as CD163B^+ ^macrophages in the upper dermis. This correlated with significantly increased mRNA levels of *αSMA*, *Emr1*, and *MCP1*. Infiltration of CD3-, CD45/B220-, and Mac3-positive cells was observed mainly in the hypodermis. Furthermore, an increased number of double-positive VE-cadherin/FSP1 cells were detected in the hypodermis only.

**Conclusions:**

This work demonstrates that Ang II induces both inflammation and fibrosis in the skin via MCP1 upregulation and accumulation of activated fibroblasts. Additionally, our data suggest that populations of these fibroblasts originate from circulating blood cells. Ang II infusion via osmotic minipumps could serve as a useful mouse model of skin fibrosis to gain new insights into pathogenic mechanisms and to test new antifibrotic therapies.

## Introduction

Systemic sclerosis (SSc) is a complex autoimmune inflammatory disorder of unknown etiology characterized by vascular alterations, activation of the immune system, and fibrosis of the skin and internal organs [1]. Vascular insufficiency and immune dysfunction manifest early in the disease and are followed by increased extracellular matrix (ECM) production as the disease progresses. Raynaud phenomenon (RP) is present in the majority of SSc patients and can precede definite diagnosis of SSc by years or even decades. In patients with SSc, RP is associated with structural abnormalities of the microvasculature and the presence of SSc-specific autoantibodies [2], indicating an early link between immune system activation and vascular injury. Fibrosis results from excessive production and accumulation of ECM components produced by activated fibroblasts, which might be triggered by cytokines and growth factors released from the infiltrating immune cells during the inflammatory stage [3]. However, interrelations between the key pathologic components of the disease are still poorly understood.

Angiotensin II (Ang II), a main component of the rennin-angiotensin system (RAS), is a vasoactive peptide that induces vascular constriction, salt and water retention, and increased blood pressure [4]. Ang II has been reported to play a critical role in renal and heart fibrosis through inflammation and upregulation of matrix deposition [5,6]. Previous studies also suggest that Ang II may be involved in the pathogenesis of skin fibrosis in SSc. It has been shown that Ang II levels are increased in the blood of SSc patients and that, in contrast to healthy skin, the Ang II precursor angiotensinogen is expressed in SSc skin [7]. Furthermore, the profibrotic effects of Ang II are mediated via the AT1a receptor in cultured human and mouse skin fibroblasts [8]. In addition, dysregulation of RAS components was shown in patients with SSc, with a prevalence of the vasoconstricting Ang II over the vasodilating Ang-(1-7), suggesting inhibition of endothelium-dependent vasodilatation and increased vasoconstriction in SSc vessels [9].

Utilization of animal models has been instrumental in delineating complex pathologic features of SSc. In the last decade, a number of new animal models became available to study mechanisms of SSc fibrosis [10-13]. The inducible models of SSc include the widely studied bleomycin and more recently established hypochlorous acid (HOCH) injection models, as well as the immune-based sclerodermatous graft-versus-host model. A growing number of genetic models are very valuable for investigating specific signaling pathways involved in fibrosis [14,15]. Whereas none of the currently available models recapitulate the complex features of SSc, they provide important insights into selected aspects of SSc pathogenesis and allow preclinical testing of antifibrotic compounds.

Angiotensin II has been widely used to investigate kidney [5], heart [6], and liver [16] fibrosis by using mouse models. However, the profibrotic potential of Ang II has not been evaluated in any model of dermal fibrosis. Given the potential involvement of Ang II in the pathogenesis of SSc, the goal of this study was to investigate the effect of Ang II on dermal fibrosis in a mouse model.

## Materials and methods

### Subcutaneous infusion of angiotensin II using ALZET osmotic minipumps

C57BL/6 mice were purchased from The Jackson Laboratory. All of the experiments were performed under the guidelines of the Boston University Institutional Animal Care and Use Committee (protocol AN-15037). Alzet osmotic miniature pumps (model 2002) delivering angiotensin II (Sigma-Aldrich, St. Louis, MO, USA) at a rate of 1,000 ng/kg/min (pressor dose) or 2,000 ng/kg/min, or PBS, were implanted subcutaneously on the backs of 8-week-old mice. After 14 days, mice were killed, and the skin surrounding the pump outlet was collected by using an 8-mm-diameter punch biopsy device.

### Gomori Trichrome staining

Gomori Trichrome staining was used to detect collagen fibers and collagen deposition in the mouse skin. The skin samples were fixed in 4% paraformaldehyde for 24 hours and then processed for paraffin embedding. Staining was performed on 8-μm-thick paraffin sections by following the manufacturer's instructions (Chromaview, Dublin, OH, USA; Gomori Trichrome blue collagen Kit S7440-19). Collagen fibers were stained blue, nuclei were stained black, and the background was stained red.

### Hydroxyproline assay

Collagen deposition was quantified by measuring total hydroxyproline content in 4-mm skin-punch biopsies obtained from PBS and Ang II infusion sites by using a previously described method with some modifications [17]. In brief, the skin samples were hydrolyzed with 6 *M *sodium hydroxide at 110°C for 12 hours. The hydrolyzate was then oxidized with oxidation buffer (one part 7% chloramine T and four parts of acetate citrate buffer) for 4 minutes at room temperature. Ehrlich aldehyde reagent was added to each sample, and the chromophore was developed by incubating the samples at 65°C for 25 minutes. Absorbance of each sample was read at 560 nm by using a spectrophometer. Results were expressed as total hydroxyproline content (in micrograms) per 0.1 g of tissue. A standard curve was performed for all hydroxyproline measurements by using known quantities of hydroxyproline.

### Quantitative RT-PCR analysis

Total RNA was isolated by using the RNeasy Fibrous Tissue Mini Kit (Qiagen, Valencia, CA, USA). Then, 1 μg of total RNA was reverse transcribed with random hexamers by using the Transcriptor First Strand Complementary DNA Synthesis kit (Roche Applied Science, Indianapolis, IN, USA) according to the manufacturer's protocol. Real-time PCR assays were performed by using the StepOnePlus Real-Time PCR system (Applied Biosystems, Foster City, CA, USA). The amplification mixture (10 μl) contained 1 μl of complementary DNA, 0.5 μ*M *of each primer, and 5 μl of SYBR Green PCR Master Mix. The primers are listed in Supplementary Table 1. Relative change in the levels of genes of interest was determined by the 2^-ΔΔCT ^method.

**Table 1:** Primers for quantitative real-time polymerase chain reaction

m-B2MG	Forward:	5'-TCGCTCGGTGACCCTAGTCTTT-3'
	
	Reverse:	5'-ATGTTCGGCTTCCCATTCTCC-3'
m-Fli1	Forward:	5'-ACTTGGCCAAATGGACGGGACTAT-3'
	
	Reverse:	5'-CCCGTAGTCAGGACTCCCG-3'

m-Col1a1	Forward:	5'-GCCAAGAAGACATCCCTGAAG-3'
	
	Reverse:	5'-TGTGGCAGATACAGATCAAGC-3'

m-Col1a2	Forward:	5'-GCCACCATTGATAGTCTCTCC-3'
	
	Reverse:	5'-CACCCCAGCGAAGAACTCATA-3'

m-COL3a1	Forward:	5'-GCCAAGAAGACATCCCTGAAG-3'
	
	Reverse:	5'-TGGACTGCTGTGCCAAAATA-3'

m-Col5a1	Forward:	5'-GGACTAGTCCGCTTTCCCTGTCAACTTG-3'
	
	Reverse:	5'-GTGGTCACTGCGGCTGAGGAACTTC-3'

m-CTGF	Forward:	5'-CTGCAGACTGGAGAAGCAGA-3'
	
	Reverse:	5'-GATGCACTTTTTGCCCTTCTT-3'

m-aSMA	Forward:	5'-CCCACCCAGAGTGGAGAA-3'
	
	Reverse:	5'-ACATAGCTGGAGCAGCGTCT-3'

m-FSP1	Forward:	5'-GGAGCTGCCTAGCTTCCTG-3'
	
	Reverse:	5'-TCCTGGAAGTCAACTTCATTGTC-3'

m-MCP1	Forward:	5'-CATCCACGTGTTGGCTCA-3'
	
	Reverse:	5'-GATCATCTTGCTGGTGAATGAGT-3'

m-CD3	Forward:	5'-AACACGTACTTGTACCTGAAAGCTC-3'
	
	Reverse:	5'-GATGATTATGGCTACTGCTGTCA-3'

m-Emr1	Forward:	5'-CCTGGACGAATCCTGTGAAG-3'
	
	Reverse:	5'-GGTGGGACCACAGAGAGTTG-3'

m-CD45/B220	Forward:	5'-AATGGCTCTTCAGAGACCACATA-3'
	
	Reverse:	5'-AGTCAGGCTGTGGGGACA-3'

TGF-β1	Forward:	5'-GCAGCACGTGGAGCTGTA-3'
	
	Reverse:	5'-CAGCCGGTTGCTGAGGTA-3

TGF-β2	Forward:	5'-CCTTCTTCCCCTCCGAAAC-3'
	
	Reverse:	5'-AGAGCACCTGGGACTGTCTG-3'

TGF-β3	Forward:	5'-AAGAAGCGGGCTTTGGAC-3'
	
	Reverse:	5'-CGCACACAGCAGTTCTCC-3'

### Immunofluorescence staining on frozen sections

For all immunofluorescence staining, skin samples were directly embedded in O.C.T. compound, flash frozen, and stored at -80°C. Staining was performed on 8-μm cryosections of mouse skin. In brief, slides were blocked with a blocking solution (3% BSA (Sigma-Aldrich), and 0.3% Triton X-100 in PBS) for 2 hours. After washing, tissue sections were incubated at 4°C overnight with primary antibodies (Table 2). Tissue sections were then washed and incubated with secondary Ab (Table 2) at room temperature for 2 hours. Coverslips were mounted by using Vectashield with DAPI (Vector Laboratories, Burlingame, CA, USA), and staining was examined by using a FluoView FV10i confocal microscope system (Olympus, Center Valley, PA, USA) at 488 nm (green), 594 nm (red), and 405 nm (blue).

**Table 2:** Primary and secondary antibodies for immunofluorescence staining

	Primary antibodies		Secondary antibodies	
Myofibroblasts	Rabbit anti-mouse αSMA Ab (Novus Biologicals, Littleton, CO); 1:100		Alexa fluor 488 donkey anti-rabbit IgG (Invitrogen, Grand Island, NY); 1:1,000	

Fibrocytes	Rat anti-mouse CD45 Ab (BD Pharmingen, San Diego, CA); 1:50	Rabbit anti-mouse FSP1 Ab (Abcam, Cambridge, MA); 1:100	Alexa fluor 594 donkey anti-rat IgG (Invitrogen); 1:1,000	Alexa fluor 488 donkey anti-rabbit IgG (Invitrogen); 1:1,000

EndoMT	Goat anti-mouse VE-cadherin Ab (Santa Cruz Biotechnology, Santa Cruz, CA); 1:300	Rabbit anti-mouse FSP1 Ab (Abcam); 1:100	Alexa fluor 488 donkey anti-goat IgG (Invitrogen); 1:1,000	Alexa fluor 594 donkey anti-rabbit IgG (Invitrogen); 1:1,000

### Immunofluorescence staining on adherent cell cultures

Human dermal microvascular endothelial cells (HDMECs) were isolated from human foreskin, as previously described [18]. Cells were cultured on bovine collagen-coated six-well plates in EBM medium supplemented with 10% FBS and EC growth supplement mix at 37°C with 5% CO_2 _in air. The culture medium was changed every other day. For immunofluorescence, cultured HDMECs grown on collagen-coated coverslips were treated with Ang II (1,000 ng/ml) for 96 hours. Control and Ang II-treated cells were fixed with 4% paraformaldehyde for 15 minutes followed by incubation with 0.15 *M *glycine for 30 minutes. Nonspecific protein binding was blocked with 3% BSA for 1 hour. Next, cells were incubated at 4°C overnight with primary antibodies: goat anti-mouse VE-cadherin (Santa Cruz Biotechnology, Santa Cruz, CA, USA), and rabbit anti-mouse FSP1 (Abcam, Cambridge, MA, USA). After washing, cell cultures were incubated with Alexa fluor 488 donkey anti-goat (Invitrogen, Grand Island, NY, USA) and Alexa fluor 594 donkey anti-rabbit (Invitrogen) antibodies for 1.5 hour. Cells were mounted on slides by using Vectashield with DAPI (Vector Laboratories) and examined by using a FluoView FV10i confocal microscope system (Olympus, Center Valley, PA, USA) at 488 nm (green), 594 nm (red), and 405 nm (blue).

### Immunohistochemistry

Immunohistochemistry was performed on formalin-fixed, paraffin-embedded skin tissue sections by using the Vectastain ABC kit (Vector Laboratories) according to the manufacturer's instructions. In brief, sections (8-μm thick) were mounted on APES (aminopropyltriethoxy silane solution)-coated slides, deparaffinized with Histo-Clear (National Diagnostics, Atlanta, GA, USA), and rehydrated through a graded series of ethanol. Endogenous peroxidase was blocked by incubation in 3% hydrogen peroxide for 30 minutes, followed by incubation with 0.15 *M *glycine for 45 minutes, and normal blocking serum for 1 hour. The sections were then incubated overnight at 4°C with antibodies against CD3 (Abcam, Cambridge, MA, USA), Mac3 (BD Bioscience, San Jose, CA, USA), CD45R (AbD Serotec, Raleigh, NC, USA), or CD163B (Epitomics, Burlingame, CA, USA), diluted 1:100 in blocking buffer, followed by incubation for 30 minutes with a biotinylated secondary antibody solution. A solution containing avidin:biotin:peroxidase complexes was applied to the sections subsequently. Immunoreactivity was visualized with diaminobenzidine (Vector Laboratories), and the sections were counterstained with hematoxylin. Images were collected by using a microscope (BH-2; Olympus, Center Valley, PA, USA).

### Statistical analyses

All data were analyzed with the Student paired *t *test. The level for statistical significance was set at *P *≤ 0.05.

## Results

### Angiotensin II increases collagen synthesis and deposition in mouse skin

**Figure 1:**
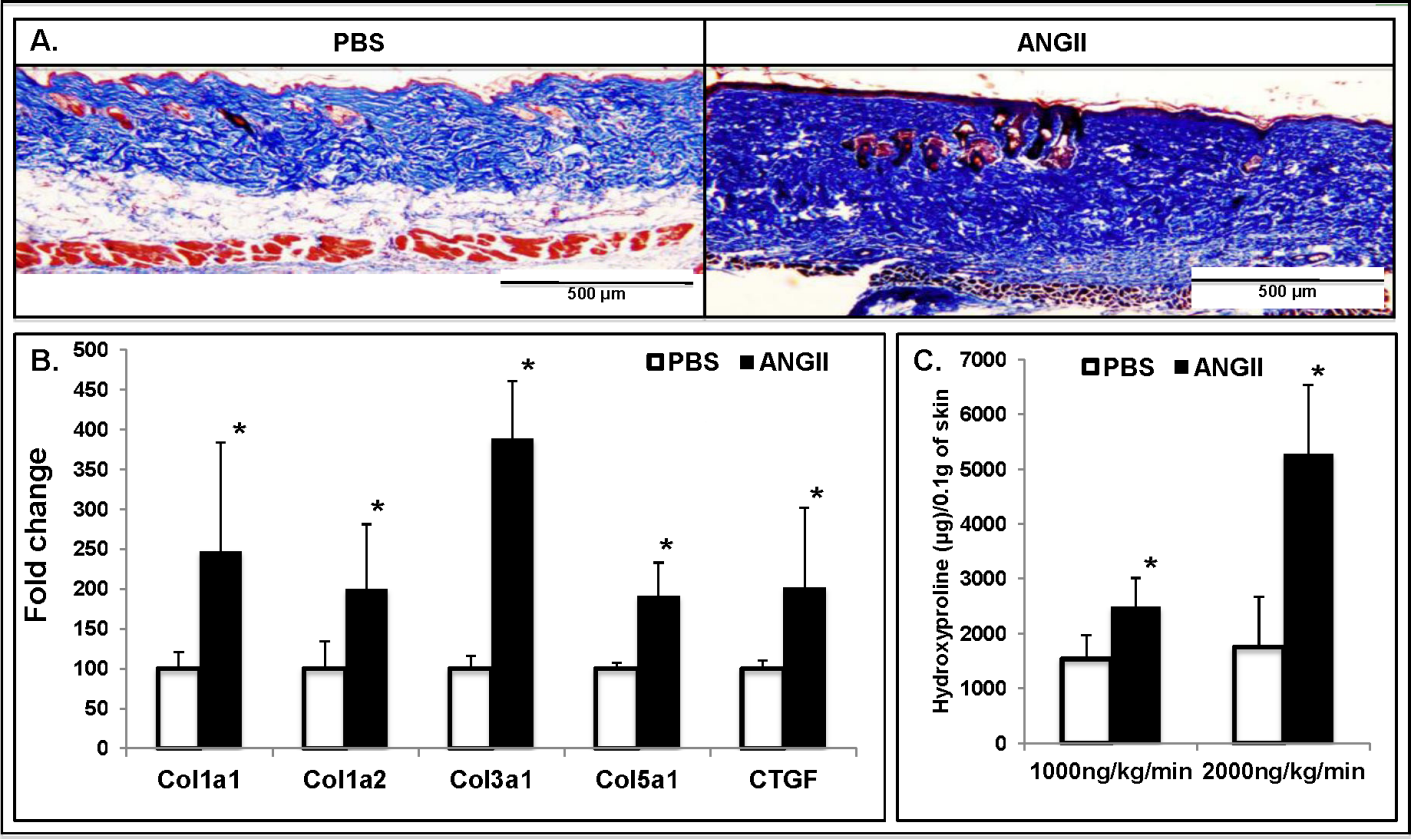
**Angiotensin II increases collagen synthesis and deposition in mouse skin**. **(A) **Gomori Trichrome staining shows a histologic evaluation of Ang II-induced lesions in C57BL/6 mice. **(B) **Real-time PCR analysis of collagen-encoding genes: *Col1a1*, *Col1a2*, *Col3a1*, *Col5a1*, along with *CTGF *(*P ≤ 0.05) in PBS-and Ang II-treated mice. **(C) **Total hydroxyproline content in PBS- and Ang II-treated mice. Values are the mean of six mice in each group; *P ≤ 0.05.

To determine whether angiotensin II can induce dermal fibrosis, Ang II at a rate of 1,000 ng/kg/min was administered continuously with subcutaneous osmotic minipumps implanted under the shaved back skin of 8-week-old C57BL/6 male mice. Treated skin was harvested at day 14, and histologic examination and collagen-content measurement assays were performed. Gomori Trichrome staining showed that collagen fibers were more closely packed in angiotensin II-treated mice compared with control mice, reflecting increased collagen deposition (Figure 1A). In addition, the fat layer of the reticular dermis was partially replaced with extracellular matrix (Figure 1A). Real-time PCR analysis showed a statistically significant increase in mRNA levels of the collagen-encoding genes: *Col1a1*, *Col1a2*, *Col3a1*, and *Col5a1*, as well as *CTGF *(2.4-fold, 1.9-fold, and 3.4-fold, 1.9-fold, and 2.1-fold, respectively; *P ≤ 0.05) in the skin of Ang II-infused mice when compared with control mice (Figure 1B). Total hydroxyproline content from the injected sites was significantly higher in Ang II (1,000 ng/kg/min)-infused skin (2,481 ± 920 μg per 0.1 g of skin) compared with control PBS-infused skin (1,534 ± 430 μg per 0.1 g of skin; *P ≤ 0.05) (Figure 1C). A higher dose of Ang II (2,000 ng/kg/min) showed a larger increase in the total hydroxyproline content of local skin (5,272 ± 1,260 μg per 0.1 g of Ang II-infused skin versus 1,748 ± 531 μg per 0.1 g of PBS-infused skin; *P ≤ 0.05) (Figure 1C). The profibrotic effects of Ang II were local, as no significant differences were observed in hydroxyproline content in distal skin with either concentration of Ang II (data not shown). Because a dose of 1,000 ng/kg/min was sufficient to induce significant dermal fibrosis, this dose was selected for further analyses.

### Angiotensin II activates the TGF-β pathway in mouse skin

**Figure 2:**
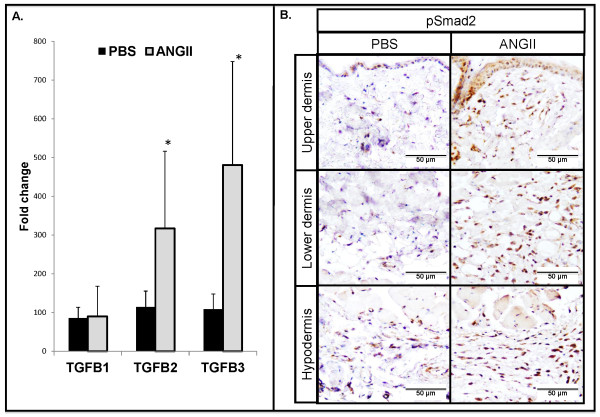
**Angiotensin II activates TGF-β signaling**. **(A) **Real-time PCR analysis of *TGF-β1*, *TGF-β2*, and *TGF-β3 *(*P ≤ 0.05) in PBS- and Ang II-treated mice. **(B) **pSmad2 staining on paraffin sections from PBS- and Ang II-infused mice. Representative images are shown from four animals per group.

Transforming growth factor-β (TGF-β) plays a pivotal role in the pathogenesis of scleroderma by activating fibroblasts and stimulating the production of ECM components [19]. To determine whether Ang II treatment activates the TGF-β pathway, we examined skin samples from the infused sites with real-time PCR and immunostaining. Real-time PCR analysis showed statistically significant increases in the mRNA levels of *TGF-β2 *and *TGF-β3 *(3.2-fold and 4.8-fold, respectively; *P ≤ 0.05) in Ang II-treated mouse skin (Figure 2A). No differences were observed in the mRNA expression of *TGF-β1 *(Figure 2A). Immunohistochemical staining of pSmad2 was performed on paraffin sections. We observed increased numbers of pSmad2-positive cells distributed throughout all dermal layers in Ang II-treated mice (Figure 2B). These data suggest that Ang II potently activates TGF-β signaling in this model.

### Angiotensin II increases the number of myofibroblasts in mouse skin

**Figure 3:**
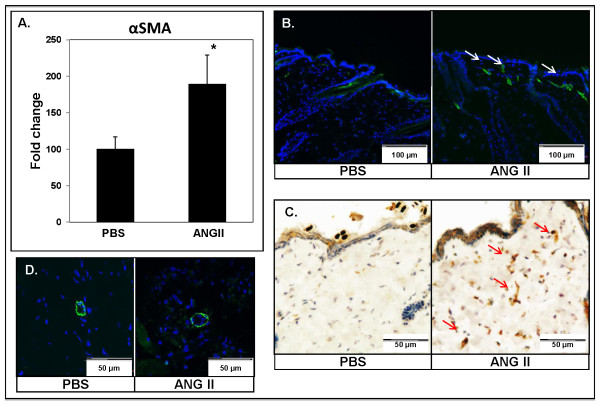
**Detection of activated fibroblasts in angiotensin II-treated skin**. **(A) **Real-time PCR analysis of *αSMA *in PBS- and Ang II-treated mice (*P ≤ 0.05). αSMA staining in the upper dermis **(B) **by immunofluorescence and **(C) **by immunohistochemistry, and in the lower dermis (**D) **by immunofluorescence, on sections from PBS- and Ang II-treated mouse skin. Arrows indicate positive staining of myofibroblasts. Representative images are shown from five animals per group.

Fibrosis is associated with accumulation of activated fibroblasts/myofibroblasts in the affected tissues. To determine whether Ang II treatment increases myofibroblast presence, skin from infused sites was examined with real-time PCR and immunostaining for αSMA. Real-time PCR analysis showed a statistically significant increase in the mRNA level of *αSMA *(1.9-fold, *P ≤ 0.05) in Ang II-treated mouse skin (Figure 3A). αSMA staining was performed on cryosections (immunofluorescence, Figure 3B and 3D) and paraffin sections (immunohistochemistry, Figure 3C) from PBS- and Ang II-treated mouse skin. Affected sites from PBS-treated mice contained few αSMA-positive cells, observed exclusively around the blood vessels and the arrector pili muscles. In contrast, affected sites from Ang II-treated mice showed an increased number of cells expressing αSMA distributed mainly throughout the upper dermis (Figure 3B and 3C), whereas in the lower dermis, positive cells were observed only around the blood vessels (Figure 3D). These data suggest that Ang II-induced myofibroblast differentiation contributes to the development of fibrosis in this model.

### Angiotensin II increases inflammation in mouse skin

**Figure 4:**
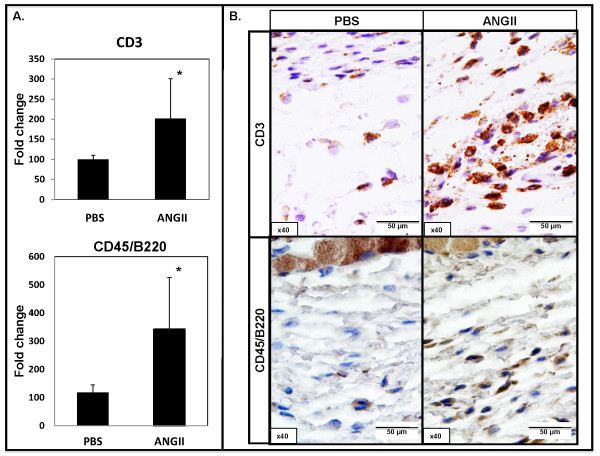
**Effect of angiotensin II on inflammation in mouse skin**. **(A) **Real-time PCR analysis of *CD3 *and *CD45/B220 *in PBS- and Ang II-treated mice (*P ≤ 0.05). **(B) **CD3 and CD45R staining on paraffin sections from PBS- and Ang II-infused mice. Representative images are shown from four animals per group.

**Figure 5:**
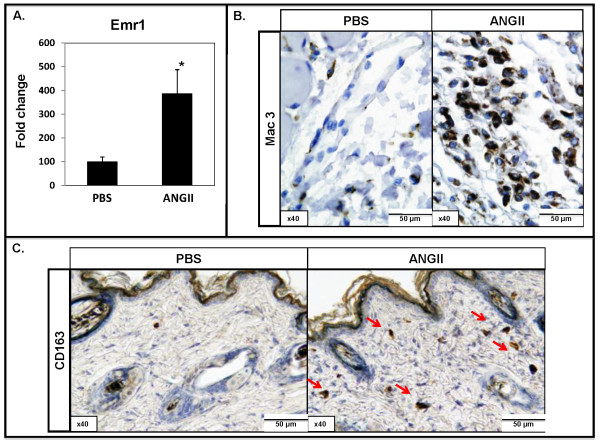
**Effect of angiotensin II on macrophage phenotype and localization in mouse skin**. **(A) **Real-time PCR analysis of *Emr1 *in PBS- and Ang II-treated mice (*P ≤ 0.05). **(B) **Mac3 and **(C) **CD163B staining on paraffin sections from PBS- and Ang II-infused mice. Arrows indicate CD163-positive cells in the upper dermis. Representative images are shown from four animals per group.

Previous studies have shown that Ang II promotes inflammation in kidney and heart models of fibrosis [5,6]. To evaluate the recruitment of inflammatory cells, skin samples from the Ang II-infused sites were examined with real-time PCR and immunostaining of immune cell markers. Real-time PCR analysis showed statistically significant increases in the mRNA levels of the T-cell marker, *CD3*, B-cell marker, *CD45/B220*, and the macrophage marker *Emr1 *(2.0-fold, 3.4-fold, and 3.6-fold, respectively; *P ≤ 0.05), in Ang II-treated mouse skin (Figure 4A and 5A). Immunohistochemical staining of CD3, CD45R, and two macrophage markers, a general marker, Mac3 [20], and a marker identifying M2 macrophages, CD163B [21,22] was performed on paraffin sections. We observed increased infiltration of CD3, CD45R, and Mac3-positive cells only in the hypodermis of Ang II-treated mice (Figure 4B and 5B). Interestingly, we observed an increased presence of CD163B-positive cells in the upper dermis of Ang II-treated mice, whereas no increase was found in the lower dermis or hypodermis (Figure 5C).

### Angiotensin II increases infiltration of fibrocytes in the upper dermis

**Figure 6:**
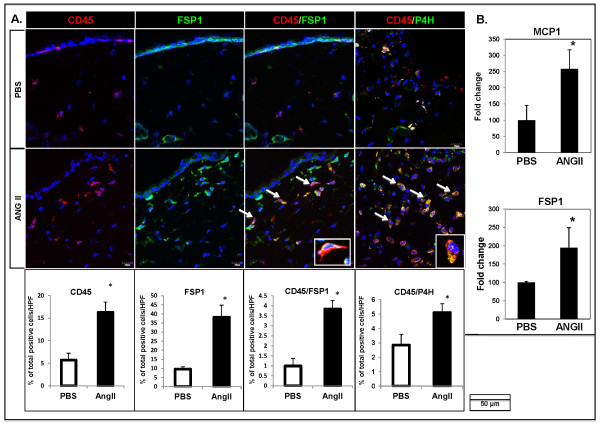
**Recruitment of fibrocytes after angiotensin II treatment**. **(A) **Immunofluorescence staining of CD45 (red) and FSP1 (green) on cryosections of skin samples from PBS- and Ang II-treated mice. Arrows indicate CD45/FSP1 and CD45/P4H double-positive cells. The bar graphs represent quantification of percentage of total positive cells/HPF (high-power field) for CD45, FSP1, CD45/FSP1, and CD45/P4H in PBS- and Ang II-treated mice (*P ≤ 0.05). Representative images are shown from four animals per group. **(B) **Real-time PCR for *MCP1 *and *FSP1 *in PBS (white bars) and Ang II (black bars)-treated mice (*P ≤ 0.05).

Circulating mesenchymal cells, fibrocytes, have been shown to infiltrate areas of inflammation and tissue damage and contribute to the fibrotic process [23]. To determine whether fibrocytes are involved in the dermal fibrosis induced by Ang II, skin cryosections were stained for the immune cell marker, CD45, and mesenchymal markers, FSP1 and P4H. Skin samples from the Ang II-injected mice showed increased numbers of the CD45/FSP1 double-positive cells (3.83% ± 0.49% versus 0.94% ± 0.37% of total number of cells/HPF; *P ≤ 0.05) distributed mostly around small vessels in the lower dermis and around the sclerotic collagen bundles in the upper dermis (Figure 6A). Similar results were observed for the CD45/P4H double-positive cells (5.11% ± 0.6% versus 2.85% ± 0.75% of cells per HPF; *P ≤ 0.05; Figure 6A). In addition, although only a few FSP1-positive cells were seen in PBS-infused skin, a significant increase in FSP1 cells was observed in the upper dermis of Ang II-infused mice (Figure 6A). We also observed a 1.55-fold increase in *FSP1 *mRNA levels in circulating cells isolated from Ang II-treated mice; however, this difference was not statistically significant (data not shown). However, mRNA levels of *FSP1 *and of the inflammatory chemokine *MCP1*, a chemoattractant for fibrocytes, were significantly elevated (1.94-fold, 2.5-fold, respectively; *P < 0.05) in Ang II-treated mouse skin (Figure 6B). No differences were observed in the mRNA expression of another fibrocyte chemoattractant, *SDF1/CXCL12 *(data not shown).

### Angiotensin II increases endothelial-to-mesenchymal transition

**Figure 7:**
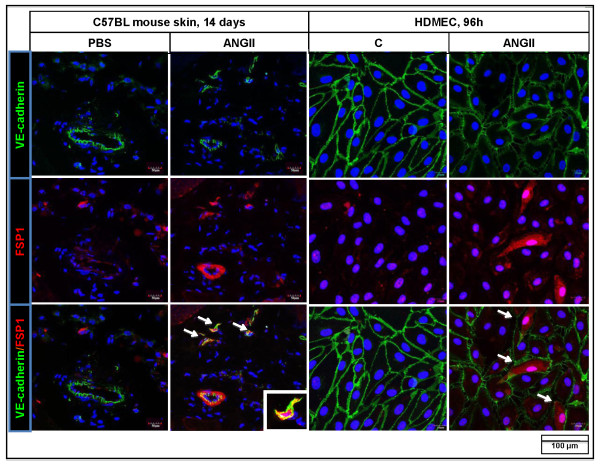
**Effect of angiotensin II on EndoMT**. Immunofluorescence staining of VE-cadherin (green) and FSP1 (red) in skin samples from PBS- and Ang II-treated mice (left panel) and HDMECs treated with 1,000 ng/ml of Ang II and control (right panel). Arrows indicate Ve-cadherin/FSP1 double-positive cells. Representative images are shown from five animals per group.

Recent studies have indicated that EndoMT can contribute to the progression of fibrotic diseases, and Ang II has been implicated in this process [24-27]. To determine whether EndoMT could serve as a source of activated fibroblasts, we performed double-fluorescence staining for VE-cadherin and FSP1 in the skin samples from Ang II- and PBS-treated mice (Figure 7A). Ang II-treated mice showed increased numbers of VE-cadherin/FSP1 double-positive cells distributed around small vessels in the lower dermis. No VE-cadherin/FSP1 double-positive cells were detected in the upper dermis. In a complementary experiment, cultured HDMECs treated with Ang II (Figure 7B) demonstrated decreased expression of the endothelial marker, VE-cadherin, and increased expression of the mesenchymal marker, FSP1.

## Discussion

The renin-angiotensin system plays a key role in organ fibrosis, including heart, kidney, lung, and liver [5,6,28,29]. In this study, we show that Ang II is also a potent inducer of dermal fibrosis in a mouse model. Consistent with its role in other organs, we provide the evidence that Ang II induces dermal fibrosis through diverse pathogenic mechanisms, including stimulation of collagen and CTGF synthesis, myofibroblast differentiation, activation of M2 macrophages, recruitment of fibrocytes, and induction of EndoMT.

Ang II infusion resulted in a dose-dependent deposition of collagen in all dermal layers that was correlated with a significant upregulation of interstitial collagen genes. Consistent with other studies, we observed activation of the TGF-β signaling pathway in response to Ang II treatment. The interaction between Ang II and TGF-β in the context of fibrosis is well characterized in many organs, including kidney and heart [5,30]. Ang II is known to act both independently and synergistically with TGF-β in promoting excessive ECM production [5,30,31]. We also observed an upregulation of CTGF, a well-known downstream mediator of the profibrotic effects of TGF-β [32]. Our data suggest that activation of resident fibroblasts is the primary mechanism responsible for extracellular matrix accumulation. Consistent with this notion, we observed increased numbers of FSP1-positive cells and increased numbers of myofibroblasts, mainly throughout the upper dermis, suggesting activation of resident fibroblasts, as well as their differentiation to myofibroblasts. Ang II may act directly on resident fibroblasts, as Ang II can upregulate both αSMA and FSP1 in cultured dermal fibroblasts (Lukasz Stawski and Maria Trojanowska, unpublished data). An increased number of myofibroblasts, correlating with increased expression of CTGF, was also observed in the bleomycin-induced model of skin fibrosis [33].

In renal and heart fibrosis models, Ang II contributes to increased infiltration of immune cells by activating the expression of the proinflammatory chemokine, MCP1 [5]. Inflammation was also shown to be closely associated with fibrosis in a bleomycin-induced model of skin fibrosis [34]. Similarly, in our study, we observed increased local infiltration of T cells, B cells, and macrophages in the hypodermis of mouse skin, which correlated with increased expression of MCP1 in response to Ang II. Recruitment of inflammatory cells by MCP1 plays an important role in skin fibrosis, as MCP1-deficient mice showed reduced fibrosis compared with WT mice in the bleomycin-induced skin fibrosis model because of the decreased recruitment of immune cells to the affected sites [35]. Importantly, we observed an increased presence of CD163B-positive cells representing a population of alternatively activated M2 macrophages in the upper dermis of Ang II-infused mice. Both classically activated macrophages (CAMs, also called M1) and alternatively activated macrophages (AAMs, also called M2) are known to play important roles in wound repair and fibrosis, either by directly releasing profibrotic cytokines or by recruiting other cell types that regulate extracellular matrix turnover [36]. In our study, M2 macrophages and myofibroblasts showed similar distribution throughout the upper dermis, suggesting a potential contribution of M2 macrophages to myofibroblast differentiation. Interestingly, colocalization of CD163B^+ ^macrophages with αSMA^+ ^myofibroblasts was recently demonstrated in the areas of glomerular and interstitial fibrosis in a model of IgA nephropathy [37]. Furthermore, CD163B^+ ^macrophages displayed strong staining for CTGF, suggesting production of this profibrotic factor [37]. Activated M2 macrophages (CD163 positive) have also been found in the skin of patients with localized scleroderma and have been implicated as a possible source of profibrotic cytokines [38].

Accumulating evidence mainly from animal models of organ fibrosis suggests that lesional myofibroblasts not only may originate from resident fibroblasts, but also may arise from circulating mesenchymal cells or from endothelial cells through endothelial-to-mesenchymal transitions. Fibrocytes have been implicated in the pathogenesis of various experimental fibrotic conditions, including bleomycin-induced pulmonary fibrosis [39], renal fibrosis [40], liver fibrosis [41], and heart fibrosis [42]. In our study, we showed an increased number of infiltrating fibrocytes (CD45/FSP1 or CD45/P4H double-positive cells) in the skin of Ang II-infused mice, which may contribute to the development of fibrosis in response to Ang II. In scleroderma, higher numbers of cells described as "collagen-producing monocytes" was observed in the peripheral blood of patients with interstitial lung disease [43,44].

It was previously reported that Ang II plays an important role in inducing EndoMT in early stages of cardiac fibrosis [45]. Increased numbers of activated fibroblasts originating from capillary endothelial cells also were shown in a bleomycin-induced lung-fibrosis model [46]. Our results show increased number of cells co-expressing the endothelial cell marker, VE-cadherin, and a mesenchymal cell marker, FSP1, in Ang II-infused mouse skin, suggesting increased EndoMT in response to Ang II. However, we have observed the presence of such cells only in the lower dermis, suggesting that they may have a limited role in this model because the majority of myofibroblasts were present in the upper dermis. However, determination of the precise contribution of the process of EndoMT to this model of dermal fibrosis will require reporter mice that would allow the lineage tracing of endothelial cells. EndoMT is induced in response to abnormal fibrillin-1 expression and chronic oxidative stress in the Tsk^+/- ^mouse, another model of SSc [47].

## Conclusions

This work demonstrates that Ang II infusion induces both inflammation and fibrosis in the skin via MCP1 upregulation and accumulation of activated fibroblasts. Additionally, our data suggest that populations of these fibroblasts originate from circulating blood cells. Elevated serum levels of Ang II found in a subset of patients with dcSS in the early stage of the disease suggest that Ang II may contribute to the pathogenesis of dcSSc, at least in a subset of patients [7]. The pathogenic features observed in the Ang II model of dermal fibrosis, such as infiltration of fibrocytes and a colocalization of myofibroblasts with the M2 macrophages, are particularly interesting, because they may help to elucidate similar processes occurring during the pathogenesis of SSc. We believe that the Ang II model of dermal fibrosis will be very useful for future mechanistic studies of SSc pathogenesis and for the evaluation of novel antifibrotic treatments.

## Abbreviations

αSMA: alpha-smooth muscle actin; Ab: antibody; Ang II: angiotensin II; APES: aminopropyltriethoxysilane; BSA: bovine serum albumin; CTGF: connective tissue growth factor; dcSS: diffuse cutaneous systemic sclerosis; EC: endothelial cell; ECM: extracellular matrix; EndoMT: endothelial-to mesenchymal transition; FBS: fetal bovine serum; FSP1: fibroblast-specific protein 1; HDMEC: human dermal microvascular endothelial cell; HPF: high-power field; MCP1: monocyte chemotactic protein-1; PBS: phosphate-buffered saline; PCR: polymerase chain reaction; RAS: renin-angiotensin system; RP: Raynaud phenomenon; SSc: systemic sclerosis; TGF-β: transforming growth factor β; WT: wild type.

## Competing interests

No conflict of interest was identified for all authors.

## Authors' contributions

LS performed all experiments and wrote the manuscript. RH performed blood analyses and contributed to manuscript writing. AB contributed to experimental design and manuscript writing. MT was the principal investigator and was involved in conception and design of the study, data analysis, and manuscript writing. All authors read and approved the manuscript for publication.
